# Extracting structure–activity relationships from patents with large language models

**DOI:** 10.1186/s12859-026-06587-w

**Published:** 2026-08-03

**Authors:** Shaolong Zheng, Gengyuan Yao, Hong Cai, Ningxuan Ma, Xiaofeng Zhang, Yuanjia Hu

**Affiliations:** 1 State Key Laboratory of Mechanism and Quality of Chinese Medicine, University of Macau, Room 1049, E12, Macao SAR, 999078 China; 2 Faculty of Computing, School of Computer Science and Technology (Shenzhen), Harbin Institute of Technology, Building L, HIT Campus, Shenzhen UniversityTown, Xili, Shenzhen, China; 3 Centre for Pharmaceutical Regulatory Sciences, Institute of Chinese Medical Sciences, University of Macau, Macao SAR, China; 4 DPM, Faculty of Health Sciences, University of Macau, Macao SAR, China

**Keywords:** Structure–activity relationships, Large language models, Pharmaceutical patent, Prompt engineering, Lead optimization

## Abstract

**Background:**

Persistent clinical failures in drug development arise from multiple factors, including imbalanced molecular properties, underscoring the need for systematic structure–activity relationship analysis to link structural modifications with optimized efficacy, selectivity, and safety of small-molecule drugs. Compared with academic literature, drug-related patent documents contain earlier and richer structure–activity relationship information that can support preclinical decision making. However, extracting structure–activity relationship information from patents is technically challenging due to ambiguous terminology, fragmented tables, dispersed compound descriptions, and the need to correctly link compound identities with activity endpoints. To address these issues, we developed PatSAR, a prompt-engineering-centered LLM workflow implemented through the ChatGPT web interface, in which finalized task-specific prompts guide document-by-document extraction of SAR information from patent PDF documents.

**Results:**

When evaluated on 214 PARP-related patents containing 8226 expert-annotated SAR entities, PatSAR achieved a recall of 79.05%, a precision of 99.65%, and an F1 score of 88.16%. In a multi-target generalization test set of 200 recent patents across ten non-PARP small-molecule targets, PatSAR achieved a recall of 85.25%, a precision of 98.97%, and an F1 score of 91.60%. In an external comparison on DocSAR-Patent-96, PatSAR achieved a Table Recall of 81.86%, compared with the reported Doc2SAR result of 74.09% on the patent subset.

**Conclusions:**

These results support the potential of large language models for patent information extraction and highlight the role of prompt engineering in improving task-specific extraction performance. The proposed PatSAR framework illustrates the promise of hybrid human–AI workflows for SAR curation and patent analysis.

**Supplementary Information:**

The online version contains supplementary material available at https://doi.org/10.1186/s12859-026-06587-w.

## Background

The development of novel small-molecule drugs remains a high-risk process, with many candidates failing during clinical development because of insufficient efficacy, toxicity, suboptimal pharmacokinetic or pharmacodynamic properties, or other clinical factors [1]. Although these failures arise from multiple causes, structure–activity relationship (SAR) analysis remains central to medicinal chemistry because it links structural modifications to biological activity, selectivity, and drug-like properties [2]. By organizing how chemical changes influence activity outcomes, SAR analysis supports lead optimization and helps researchers interpret molecular design choices during preclinical development [2, 3].

Academic publications have traditionally served as an important source of SAR information, but they often provide delayed and incomplete coverage of compound–activity data, capturing only approximately 20% of disclosed chemical entities compared with patents [4]. By contrast, drug-related patent documents have been reported to disclose SAR data two to three years earlier and to contain three to five times more compound–activity pairs, including structural optimization examples that may not be fully reported in journal articles [4–6]. However, the practical use of patent-derived SAR data remains challenging. Patent documents often distribute relevant information across activity tables, synthetic examples, compound descriptions, claims, and assay Sections [5]. Terminology may also vary across jurisdictions, languages, applicants, and patent drafting styles. As a result, extracting patent-derived SAR data requires not only recognizing compounds and activity values, but also correctly linking compound identity information with the corresponding assay endpoints and measurements [5]. This gap highlights the need for computational frameworks that can extract and structure patent-derived SAR data, thereby supporting more efficient preclinical decision making.

Existing approaches only partially address this problem. Manual curation remains important for high-quality SAR interpretation, but it is time-consuming and difficult to scale across large patent collections [2, 3, 5, 6]. Curated databases and patent-mining platforms provide useful structured resources, but their coverage may be incomplete, delayed, or limited to predefined data sources and formats [6–10]. Semi-automated tools and commercial patent analytics systems can reduce manual workload, particularly for well-structured tables, but they may be less effective when SAR information is embedded in narrative text, dispersed across sections, or presented through nonstandard formatting [11–16]. Recent large language models (LLMs) based and document-level extraction studies [17–20] have further shown that large language models can support structured information extraction from scientific documents and patents [21, 22]. Nevertheless, reliable patent-derived SAR extraction remains difficult because the task requires accurate compound–activity linkage, conservative handling of missing or unsupported information, and structured outputs that can be reviewed by domain experts [23].

Recent advances in artificial intelligence, particularly LLMs, provide new opportunities for processing long technical documents and extracting structured information from heterogeneous text, tables, and images [24, 25]. For patent-derived SAR extraction, the potential value of LLMs lies in their ability to interpret information across document sections and to generate structured summaries from complex inputs. However, a general request to “extract SAR data” is insufficient for this task. Patent SAR extraction requires explicit operational rules defining what counts as an SAR entity, how compound identifiers or structural descriptions should be linked to activity endpoints, which chemical entities should be excluded, how unsupported or unavailable information should be marked, and how final outputs should be organized.

These challenges motivated the use of task-specific prompt engineering rather than a general extraction request. In this study, prompt engineering was used to translate patent SAR extraction into explicit operational instructions, including SAR entity definitions, compound–activity linkage rules, exclusion criteria for non-SAR chemical entities, conservative handling of unsupported information, and standardized SAR table output. Prompt strategies such as task decomposition, stepwise checking, and few-shot examples were incorporated to guide the model through the sequential operations required for SAR extraction from patent PDFs [26–30]. These strategies were implemented at the prompt level and did not involve parameter-level model training or fine-tuning.

**Fig. 1:**
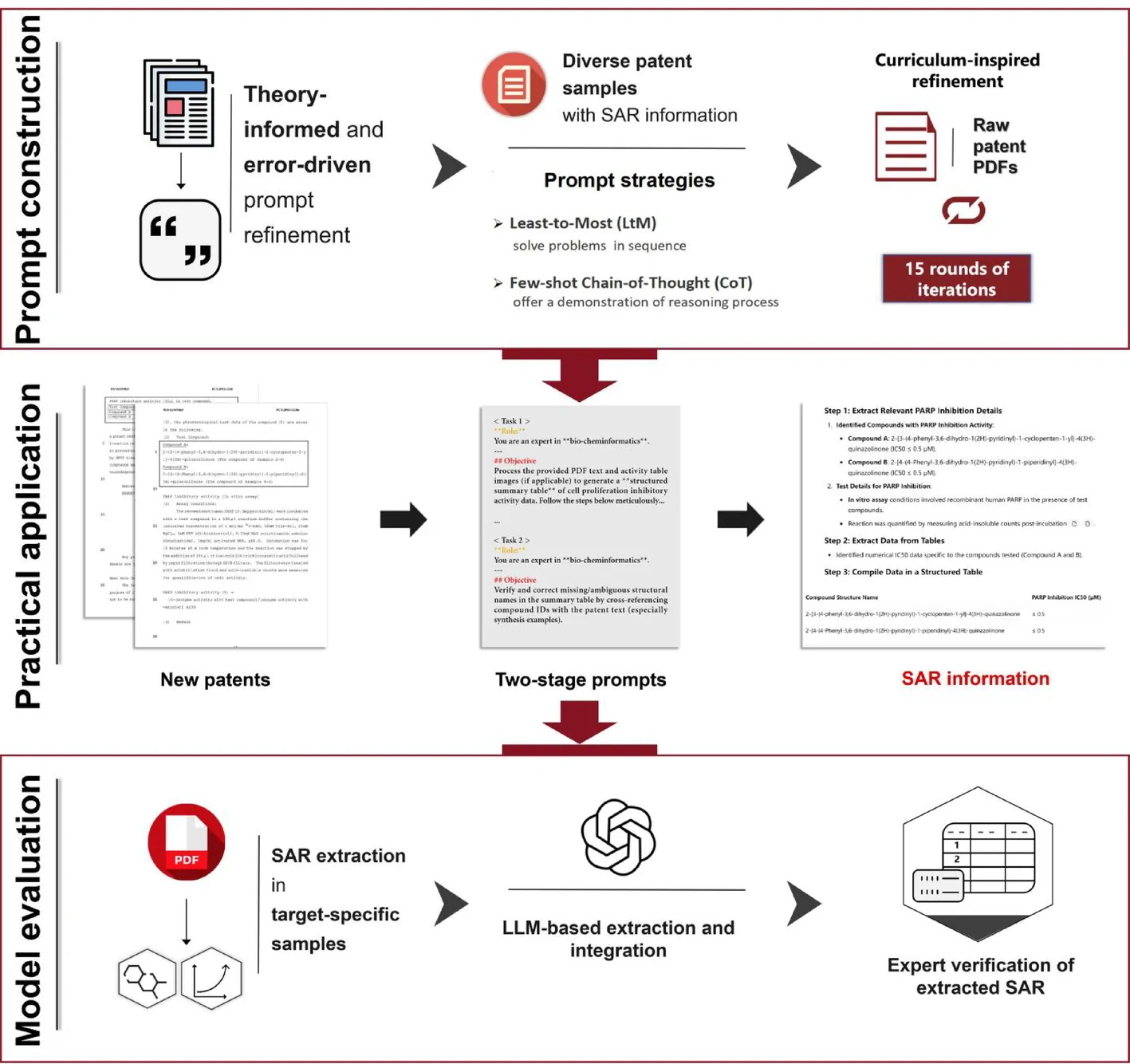
Research workflow for SAR extraction from patents using prompt engineering. PatSAR includes three components: prompt construction, practical application, and model evaluation. In prompt construction, theory-informed and error-driven refinement was conducted on diverse patent samples using least-to-most prompting, few-shot chain-of-thought prompting, and curriculum-inspired iteration. In practical application, new patent PDFs were processed with two-stage prompts to extract compound–activity information and complete unresolved structural entries, producing structured SAR tables. In model evaluation, extracted SAR information was compared with expert-verified reference annotations across target-specific patent datasets

Here, we developed PatSAR, a prompt-based LLM workflow for extracting SAR information from patent PDF documents. The workflow was designed to convert dispersed patent content into structured SAR tables by identifying compound identities, extracting associated activity endpoints and values, excluding non-SAR entities, and linking information across tables and narrative sections. As summarized in Fig. 1, PatSAR consists of three major components: task-specific prompt construction through theory-informed and error-driven refinement, document-level application to new patent PDFs using a two-stage extraction prompt, and evaluation against expert reference annotations across target-specific and external patent datasets. Through this design, the study aimed to assess whether task-specific prompt engineering can adapt a general-purpose LLM interface to patent-derived SAR extraction, thereby supporting human–AI curation of medicinal chemistry information and generating structured SAR evidence that may inform lead optimization and candidate prioritization in preclinical research.

## Methods

### Model selection

The extraction of structure–activity relationship (SAR) data from unstructured patent documents typically relies on time-consuming manual annotations or automated extraction techniques. These methods are resource-intensive, often resulting in high costs. In response to these challenges, we considered leveraging LLMs to automate this process. Initially, we considered several candidate models, including Claude, Kimi, Gemini, and GPT, with reference to the Document Arena ranking on the LMArena platform [31]. This comparison was based on factors such as the models’ ability to handle long documents, their contextual reasoning capabilities, and their performance on document-oriented tasks, particularly in extracting structured data from patent texts.

ChatGPT-5 was selected for subsequent development because it provided a practical combination of long-document processing, multimodal input handling, structured-output generation, and accessibility through a web-based interface. While other models such as Claude and Kimi also showed strong performance in general document-processing tasks, GPT’s multimodal capabilities, particularly its ability to process patent PDF inputs containing text, tables, and page-level visual information [24, 25], made it a suitable implementation platform for extracting SAR information from patents. Furthermore, GPT-based models have been used in document-level and chemistry-related information extraction workflows, supporting their feasibility for structured data extraction from complex scientific documents [17–20]. In the Results section, we further contextualize PatSAR through a functional comparison with related chemical information extraction tools, patent analytics platforms, and curated bioactivity resources.

### Prompt generation and iteration

Prompt design was a central component of PatSAR because patent-based structure–activity relationship (SAR) extraction requires the model to identify relevant compounds, locate corresponding bioactivity data, and integrate information distributed across tables, examples, descriptions, and claims. Previous studies have suggested that prompt engineering can improve the performance of large language models by defining task objectives, reasoning procedures, constraints, and output formats without modifying model parameters [32–34]. Therefore, we used prompt engineering to convert SAR extraction into a set of explicit and reproducible instructions. During prompt iteration, the main prompt-engineering elements included task decomposition consistent with least-to-most prompting [27, 28], stepwise reasoning instructions consistent with zero-shot chain-of-thought prompting [29], and few-shot examples introduced in later versions to clarify SAR entity boundaries, inclusion and exclusion criteria, and output-format requirements [30]. These elements were incorporated at the prompt level and did not involve parameter-level model training.

In this study, an SAR entity was operationally defined as a compound-specific activity record that contributed to a structure–activity relationship and could be linked to a patent-reported compound identifier, structural name, or structural description. The minimum required information was a compound identifier or IUPAC name together with an explicitly reported activity endpoint. Examples included IC50, Ki, EC50, Kd, percentage inhibition, activity grade, selectivity-related activity values, or other patent-reported bioactivity measurements, together with their units and assay context when available. If one compound was reported with multiple activity endpoints or assays, each compound–endpoint association was counted as a separate SAR entity. Dose–response curves or thermodynamic profiles were not digitized or reconstructed as curve-level data; they were included only when the patent provided explicit extractable summary parameters, such as an IC50, Kd, or thermodynamic value linked to a specific compound. General assay descriptions, synthetic intermediates without bioactivity data, unlinked activity values, and unsupported inferred relationships were excluded.

Prompt generation began with a deliberately simple seed instruction written by the authors, denoted as P1, which asked the model to extract SAR-related information from a prototype patent. This initial prompt did not provide an operational definition of SAR entities, inclusion or exclusion criteria, representative examples, stepwise reasoning instructions, or a standardized output schema. Thus, P1 served as a baseline prompt for identifying the limitations of direct task instruction. Its outputs were compared with expert-annotated SAR information, and recurrent failure modes, including incomplete activity extraction, inconsistent compound–activity matching, inappropriate inclusion of non-SAR entities, and unstable table formatting, were used to guide subsequent prompt refinement. Subsequent prompt revisions were also made by the authors, using model outputs and expert annotations as feedback.

Based on the errors observed with P1, subsequent prompt iterations incorporated several prompt-engineering elements. These included task decomposition consistent with least-to-most prompting, stepwise reasoning instructions consistent with zero-shot chain-of-thought prompting, and few-shot examples introduced in later versions to clarify SAR entity boundaries, inclusion and exclusion criteria, and output-format requirements. These elements were incorporated at the prompt level and did not involve parameter-level model training. The refined prompt defined the expected output as a structured SAR table containing compound identifiers, structural names, activity types, activity values, and units. It instructed the model to extract only compounds associated with relevant biological activity data and to exclude reagents, intermediates, solvents, and other non-SAR chemical entities. To reduce unsupported inference, the model was instructed to assign “N/A” when required information could not be directly obtained or reliably inferred from the source document. The extraction process was organized into two sequential stages: the first focused on identifying compound–activity associations, and the second revisited unresolved structural entries by examining examples, preparation procedures, and compound description sections. General analytical instructions were also supported by the GPT memory function; this configuration did not involve model fine-tuning, and its role as persistent task-level guidance is discussed further in the Discussion section and Supplementary Materials.

Prompt refinement followed a curriculum-inspired, difficulty-guided protocol. Here, “curriculum” refers to the easy-to-hard organization of prompt evaluation cases rather than parameter-level training of the LLM. Before prompt iteration, the prompt development patents were arranged in an approximate order of extraction difficulty based on observable document features, including whether SAR data were presented in explicit tables, whether compound identifiers were directly linked to activity values, whether structural names were provided in the same section, the extent of cross-sectional information fragmentation, table complexity, and language or formatting heterogeneity. This arrangement allowed early iterations to establish the basic extraction schema on relatively explicit SAR disclosures, while later, more complex cases exposed additional failure modes that guided targeted prompt revisions. Let D1–Dn denote these prompt development patents. The seed prompt P1 was first evaluated on relatively simple patents with explicit SAR tables and direct compound–activity mappings. If a prompt Pi did not show major new error categories on Di, it was retained for evaluation on the next document. If substantial extraction failures occurred, the errors were analyzed, and the prompt was updated to Pi + 1 by adding corrective rules, clarifying definitions, tightening extraction boundaries, or standardizing the output schema. Later iterations introduced more complex cases, including patents with dispersed activity descriptions, incomplete compound–activity mappings, complex tables, multilingual terminology, and fragmented cross-sectional information.

During prompt development, performance was monitored primarily using the false-negative rate (FNR) of SAR entity extraction, using Eq. (1):

1 $$\mathrm{F}\mathrm{N}\mathrm{R}=\frac{\mathrm{F}\mathrm{N}}{\mathrm{F}\mathrm{N}+\mathrm{T}\mathrm{P}}$$

where true positives (TPs) were SAR entities correctly extracted by the model, and false negatives (FNs) were SAR entities present in the reference annotation but missed by the model. FNR was selected as the principal development-stage indicator because the primary objective of prompt refinement was to improve extraction completeness. In early prompt versions, the dominant failure mode was the omission of SAR entities that were present in the reference annotations; therefore, FNR directly quantified the proportion of reference SAR entities missed by the model. False positives were also recorded during development to monitor unsupported or incorrectly linked outputs. After prompt finalization, the framework was evaluated on independent test sets using precision, recall, and F1 score.

Through 15 rounds of prompt evaluation and error-driven revision, the initial seed prompt was refined into the finalized version used for subsequent analyses. Prompt iteration was stopped when no major new error categories were observed and later modifications mainly involved wording clarification or output-format standardization. To avoid test-set leakage, the finalized prompt was determined before evaluation and was not further modified using results from the core test set, generalization test set, or external comparison set.

### Expert reference construction and verification

For prompt development and assessment, the expert reference was constructed by directly marking SAR-containing regions in the original patent PDFs. Eligible regions included tables, text blocks, or other document regions that explicitly reported a tested compound, biological target, activity metric, value, and unit.

Each patent PDF was independently reviewed by two researchers familiar with biological activity data, chemical structures, and patent documents. During the initial marking step, the two researchers worked separately and did not refer to each other’s annotations. They marked SAR-containing regions, including activity tables, biological assay descriptions, and other patent sections where explicit compound–target–activity data were reported. The third researcher then compared the two sets of marked regions, summarized regions marked by both researchers and regions marked by only one researcher, and calculated region-level inter-annotator agreement. The third researcher also reviewed all discrepant regions and a random subset of agreed regions against the original PDF content to confirm whether the marked regions met the predefined eligibility criteria.

Region-level inter-annotator agreement was calculated based on whether the two researchers marked the same SAR-containing source regions within each patent PDF. Regions were considered matched when they referred to the same activity table, assay description, or text block reporting the same compound–target–activity information, even if the marked boundaries were not identical. Using this region-level matching criterion, the two independent reviewers marked the same underlying SAR-containing source region for 97.4% of the source regions identified during the initial double-review process. This high agreement reflected the region-level nature of the task, in which most eligible regions contained explicit activity tables or text passages reporting compound–target–activity information, rather than requiring subjective field-level interpretation. Most disagreements involved complex activity tables, patents without structured SAR tables in which activity information was reported only in short text passages, or source regions where the SAR relevance required further checking.

All discrepant regions were then reviewed against the original patent PDFs by the third researcher. When both annotations referred to the same SAR-containing table or text passage, they were merged into a single reference source region. Regions were retained only when they clearly reported a tested compound, biological target, activity metric, value, and unit. Regions that described only synthesis or compounds without activity data were excluded. This adjudication step produced the final set of expert-marked SAR source regions used as the reference evidence for model assessment.

During model assessment, each model-extracted SAR record was checked against the final expert-marked source regions and the original PDF content. A model output was counted as correct only when the compound, target, activity metric, value, and unit could be directly verified from the corresponding source region. To assess reference completeness, we additionally reviewed model-extracted records that were not covered by the initial marked regions but appeared potentially valid. Among 86 such records, 12 were confirmed to correspond to SAR evidence regions that had been missed during the initial marking and were added to the final reference set, whereas 74 were excluded because they represented ambiguous assignments, non-SAR information, or model misinterpretations. The initial reference evidence set contained 612 SAR source regions, and 12 additional regions were added after reverse checking. Based on this process, the estimated omission rate of the initial reference evidence set was 1.92%.

### Prompt development set: PatSAR-Dev-50

To develop and refine the prompt strategy, we constructed a prompt development set, termed PatSAR-Dev-50. This set was designed to examine prompt behavior across patents with different document formats and SAR reporting patterns, rather than to serve as a benchmark for final performance estimation. Accordingly, PatSAR-Dev-50 was used only during prompt development and was excluded from all subsequent test-set evaluations.

PatSAR-Dev-50 was constructed by first identifying clinically or commercially relevant small-molecule drugs from publicly available information. The names of these drugs were then used to retrieve related patent documents from public patent databases, primarily the European Patent Office and Google Patents. In this study, drug-related patent documents refer to patent documents associated with small-molecule therapeutic agents or their analogues and containing information relevant to medicinal chemistry, such as compound preparation, structural optimization, bioactivity data, or SAR descriptions. Candidate patents were then manually screened for the presence of explicit SAR-relevant information, including compound–activity tables, dose–response data, bioactivity values linked to compound identifiers, or textual descriptions connecting structural modifications with biological activity. Patents that did not contain actionable SAR information were excluded.

The final set consisted of 50 drug-related patent documents covering diverse therapeutic areas, target classes, publication years, languages, and document layouts. No additional filtering was applied according to the apparent difficulty of extraction, table structure, chemical scaffold type, or formatting style once the inclusion criteria were met. The original PDF formats, including text, tables, and chemical structure images, were retained to approximate real-world patent analysis conditions.

### Core test set: PatSAR-Test-214

The performance of the finalized prompt was evaluated using an independent expert-annotated test set. For this purpose, we constructed PatSAR-Test-214, which consisted of 214 patents related to poly(ADP-ribose) polymerase (PARP). This set was constructed for evaluating SAR extraction from patents containing explicit SAR-relevant information and was not intended to estimate performance on no-SAR or irrelevant patent documents.

PARP was selected as the core evaluation target because it is a well-established small-molecule drug target in oncology and has generated a substantial patent landscape containing diverse SAR disclosures. These patents include compound–activity tables, scaffold optimization examples, dose–response data, and activity descriptions distributed across different sections of patent documents. Therefore, PARP patents provided a suitable corpus for evaluating whether the prompt could extract SAR information from heterogeneous but target-consistent patent materials.

PatSAR-Test-214 was not designed as a random sample of all PARP-related patents. Instead, the patents were drawn from an internally curated collection of PARP patent documents that had undergone prior expert review. This design was adopted because the purpose of this dataset was entity-level performance evaluation rather than estimation of the overall distribution of SAR reporting patterns in the PARP patent landscape. The availability of expert-marked SAR source regions allowed each model-generated SAR entry to be verified against the original patent evidence.

For each patent, the expert reference was established using the source-region marking and verification procedure described above. The finalized PatSAR prompt was then applied to all PatSAR-Test-214 patent PDFs using ChatGPT-5. Model outputs were evaluated at the SAR-entity level by checking each extracted record against the expert-marked SAR source regions and the original PDF content. Correctly extracted SAR entities were counted as true positives; reference SAR entities missed by the model were counted as false negatives; and incorrectly extracted non-SAR entries, mismatched compound–activity relationships, or unsupported structural or activity completions were counted as false positives.

### Generalization test set: PatSAR-GenTest-200

To evaluate whether the finalized prompt could maintain its extraction performance beyond the PARP-focused core test set, we constructed an additional generalization test set, termed PatSAR-GenTest-200. This dataset was designed to include patents from multiple non-PARP small-molecule targets and to assess prompt performance across broader target classes and patent writing styles.

Ten targets were selected to cover different pharmacological mechanisms and therapeutic areas [35, 36]: ALK, BTK, CDK, c-MET, EGFR, GLP-1, HER2, IL-17A, KRAS, and PD-L1. These targets were chosen based on research activity, therapeutic relevance, and the availability of recent small-molecule patent disclosures containing SAR information [37, 38]. For each target, candidate patents published between January 1, 2020 and August 1, 2025 were retrieved from Google Patents using target-specific search terms, pharmacological descriptors, and SAR-related activity terms. After randomized selection and eligibility screening, 20 SAR-containing patents were included for each target, resulting in 200 patents in PatSAR-GenTest-200. The detailed search strategy and randomization script are provided in the GitHub repository described in the Availability of data and materials section.

For all included patents, SAR information was extracted through human expert review before model evaluation to establish reference annotations. These annotations included compound identifiers, available structural names or descriptions, activity types, activity values, and units when reported in the source documents. Model outputs were then compared with these annotations at the SAR-entity level using the same evaluation criteria as in PatSAR-Test-214. This dataset allowed us to examine whether prompt performance was retained across non-PARP targets and more diverse recent patent formats, thereby complementing the PARP-focused core test set.

### External comparison: DocSAR-Patent-96

To compare PatSAR with a recently reported SAR extraction framework, we used the patent subset of the DocSAR-200 benchmark from the Doc2SAR study [23]. DocSAR-200 contains both research articles and patent documents, and the Doc2SAR study reported performance separately for the patent and research article subsets. Because the present study focuses on SAR extraction from patent PDF documents, we restricted the external comparison to patent documents to maintain consistency in document type and input modality.

The available patent documents were extracted from the DocSAR-200 benchmark materials, yielding 96 available patent PDFs, which were termed DocSAR-Patent-96 in this study. This subset was used as an external benchmark independent of our prompt development set, core test set, and generalization test set. We also checked the patent publication numbers and confirmed that none of the 96 patents in DocSAR-Patent-96 overlapped with PatSAR-Dev-50, PatSAR-Test-214 or PatSAR-GenTest-200. Among the available patent PDFs, no additional filtering was applied according to patent office, language, target class, table format, or apparent extraction difficulty.

For each document in DocSAR-Patent-96, PatSAR was applied using the finalized prompt and ChatGPT-5, consistent with the model version used for the core and generalization evaluations. To align the evaluation with the Doc2SAR study, performance was measured using Table Recall, defined as the proportion of SAR table rows for which the molecule and all associated activity values were completely and correctly extracted and linked. This metric was selected because it directly reflects complete row-level SAR recovery and permits comparison with the patent-level results reported for Doc2SAR [23].

### Summary of datasets

For the datasets constructed in this study, the main characteristics are summarized in Table 1. The table reports dataset size, SAR entity counts, publication period, language composition, patent jurisdictions, and patent status. These variables were included to describe the composition and heterogeneity of the internally constructed patent datasets, particularly factors that may influence patent-text interpretation, document layout, and SAR information extraction. Detailed distributions by language, jurisdiction, patent status, IPC/CPC subclasses, and applicant or assignee are provided in Supplementary Tables S1–S4.

**Table 1:** Main characteristics of the datasets used in this study

Dataset	Patent PDFs, n	SAR entities, n	Publication period	Languages	Jurisdictions	Patent status
PatSAR-Dev-50	50	1392	2005–2024	EN, CN	WO, CN, US	Granted: 0; applications: 50
PatSAR-Test-214	214	8226	1997–2023	EN, CN, DE, JP, KR, etc	WO, US, JP, CN, etc	Granted: 197; applications: 17
PatSAR-GenTest-200	200	16826	2020–2025	EN, CN, JP, KR, ES, etc	CN, WO, US, TW, EP, etc	Granted: 39; applications: 161
DocSAR-Patent-96	96	N/A*	2005–2024	EN, CN, JP, KR, ES, etc	US, CN, AU, EP, CA, ES, JP, etc	Granted patents: 52; patent application publications: 44

The table summarizes the internally constructed datasets used for prompt development, core evaluation, and generalization testing, together with the external DocSAR-Patent-96 subset used for comparison. Reported variables include the number of patent PDF documents, the number of expert-annotated SAR entities, publication period, language composition, patent jurisdictions, and inferred patent status. Patent status was summarized as granted patents or patent applications based on available publication metadata

*DocSAR-Patent-96 was evaluated using table-level Table Recall rather than SAR-entity-level annotations

## Results

### Model and tool comparison for patent-based SAR extraction

We first screened candidate LLMs for patent-based SAR extraction by referring to Document Arena, an external leaderboard that ranks AI models for document analysis and long-content reasoning. In the displayed ranking used for this study, the leaderboard included 21 models and 109,664 votes. Claude models occupied the highest positions, whereas GPT, Kimi, and Gemini were also represented among the leading models. Specifically, GPT-5.4 ranked sixth with a score of 1482 ± 9, while Kimi-k2.6 and Gemini-3.1-pro-preview ranked eighth and tenth, respectively (Table 2). These results suggested that GPT belonged to the leading group of document-oriented models and supported its inclusion as a candidate platform for patent-based SAR extraction.

**Table 2:** External document-analysis ranking used to contextualize candidate LLMs for patent-based SAR extraction

Model family	Representative model in document arena	Rank	Score	Context length
Claude	claude-opus-4–6-thinking	1	1528 ± 10	1 M
GPT	gpt-5.4	6	1482 ± 9	1.1 M
Kimi	kimi-k2.6	8	1459 ± 19	256 K
Gemini	gemini-3.1-pro-preview	10	1451 ± 7	1 M

The table summarizes representative model families considered during model selection, using the Document Arena ranking from the LMArena platform as an external reference for document-oriented model capability. Rank and score values were taken from the leaderboard at the time of access. Context length indicates the maximum context window reported for each representative model

GPT was selected as the implementation platform because it allowed us to implement the complete workflow required in this study, including patent PDF upload, multimodal document interpretation, structured SAR table generation, memory-supported task instructions, and repeated prompt refinement within the ChatGPT environment. These functions were necessary for applying a prompt-based extraction workflow to patent documents containing narrative descriptions, tables, and chemical structure information.

After selecting an LLM-based implementation platform, we further compared PatSAR with existing chemical information extraction tools, patent analytics platforms, and curated bioactivity resources (Table 3). This comparison focused on functional coverage rather than uniform quantitative performance, because these tools differ substantially in input format, access mode, output type, and intended use.

**Table 3:** Functional comparison of PatSAR with related tools

	SureChEMBL 2.0 [11]	ChemCurator [12]	SciBite (TERMite / Chat) [13]	GOSTAR / Reaxys [14]	PatSight [16]	Uni-finder	PatSAR
Overall positioning	Patent entity recognition and database integration	Desktop structure extraction tool	Biomedical text-mining suite	Curated bioactivity database	Patent analytics (tabular SAR)	Patent analytics (tabular SAR)	Prompt-based workflow proposed in this study for SAR extraction from patent PDFs
Primary functionality	Extraction of disease, target, and compound names from patents; no activity data	Extraction and visualization of chemical structures from documents	Term-level entity recognition and semantic search	Curated SAR and bioactivity databases	Extraction of SAR data from patent tables	Extraction of SAR data from patent tables	Full-text SAR extraction via LLM and prompt engineering
Data source coverage	USPTO, WIPO, EPO, JPO, CNIPA	User-uploaded documents (EN, CN, JP)	Scientific text corpora	Proprietary curated data	User-uploaded patent documents	User-uploaded patent documents	User-uploaded patent documents
Extraction of chemical structures	✓ (preprocessed)	✓ (on-demand)	Partial	✓ (curated)	✓ (tabular)	✓ (tabular)	✓ (textual + tabular)
Extraction of bioactivity / SAR data	✗	✗	✗	✓ (curated)	✓ (tabular SAR only)	✓ (tabular SAR only)	✓ (from text and tables)
Textual context comprehension	✗	Limited (structure mentions)	✓ (term-level)	✗ (structured Database only)	Partial	Partial	✓
Integration of SAR	✗	✗	✗	✓ (curated)	Partial	Partial	✓
Real-time / On-demand processing	✗ (preprocessed database)	✓	✓ (API / cloud)	✗ (curated data)	Limited (paid tier)	Limited (restricted)	✓ (real-time inference)
Accessibility	Public	Restricted (upon request)	Restricted (upon request)	Commercial license	Restricted (login required)	Restricted (upon request)	Restricted (login required)
Supported languages	Depending on patent source	EN, CN, JP	Not mentioned	Not mentioned	Multilingual	Multilingual	Multilingual
Customization / Extensibility	✗	Limited	✓	✗	✗	✗	✓ (prompt configurable)

Representative tools and resources were compared by function, input coverage, output type, accessibility, and extensibility

Overall, most existing tools addressed one or more components of SAR extraction, such as chemical entity recognition, structure extraction, curated bioactivity retrieval, or tabular patent analytics. However, fewer tools were designed to process user-provided patent PDFs and integrate structural information with activity values from both textual descriptions and tables. PatSAR was therefore positioned as a prompt-based workflow for end-to-end SAR table generation from patent documents, rather than as a replacement for curated databases or specialized cheminformatics platforms. It can be used through a web-based LLM interface without local model deployment, but access remains dependent on login requirements, model availability, and platform-specific usage limits.

### Prompt refinement

Prompt refinement was performed using PatSAR-Dev-50, the prompt development set constructed to expose the prompt to different patent formats and SAR reporting patterns. This process converted a direct extraction instruction into a structured two-stage SAR extraction prompt. The initial seed prompt, P1, asked the model to extract SAR-related information from a patent document but did not define SAR entities, specify inclusion or exclusion criteria, impose an output schema, or provide rules for unavailable information. When applied to patents in PatSAR-Dev-50, this direct instruction produced recurrent errors, including missed activity values, inconsistent compound-activity linkage, inappropriate inclusion of non-SAR entities, and unstable table formatting. The specific prompt-engineering strategies used during refinement and their effects on model outputs are discussed further in Discussion.

Based on these error patterns, the prompt was progressively revised to include explicit task constraints. The finalized prompt first assigned the model a domain-specific role and defined the task as extracting SAR information from patent documents. It then instructed the model to identify compound identifiers and associated bioactivity data from summary tables, examples, and textual descriptions, while excluding reagents, intermediates, starting materials, and compounds without reported bioactivity values. The output of this first stage was a preliminary SAR table containing compound identifiers, available structural names, activity types, activity values, units, and unavailable entries marked as “N/A.” In the second stage, the prompt instructed the model to revisit entries with missing or ambiguous structural information by searching examples, preparation procedures, synthesis descriptions, and compound description sections. Structural information was updated only when supported by the patent text; otherwise, “N/A” was retained. Additional output constraints required preservation of original activity units and activity types, separation of different assays or targets, and avoidance of unsupported inference. The complete prompt text is provided in Box 1.

**Box 1:**
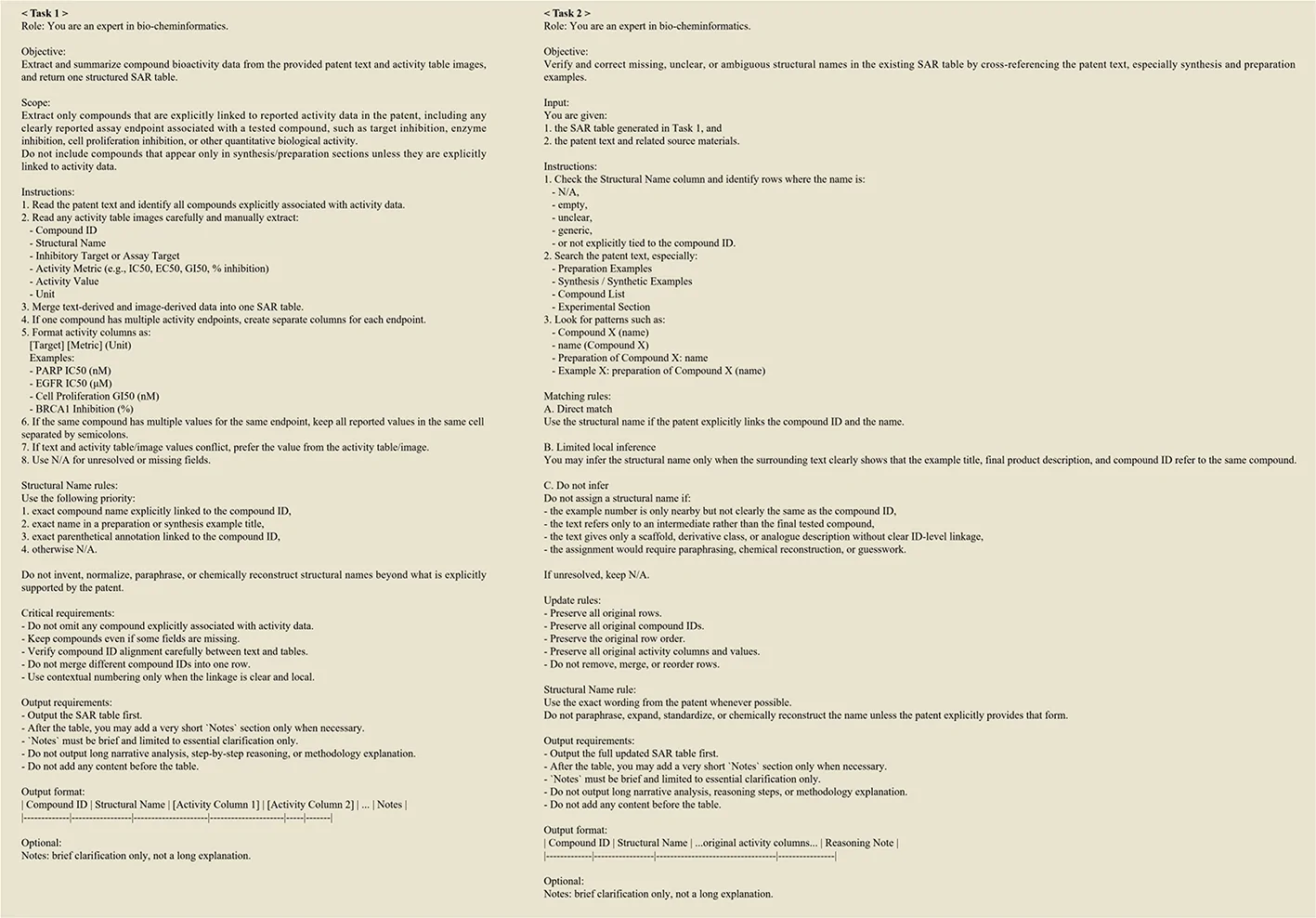
Finalized prompt used for PatSAR extraction. The prompt divides patent SAR extraction into two sequential stages to reduce task complexity, improve output consistency, and support structured SAR table generation

**Fig. 2:**
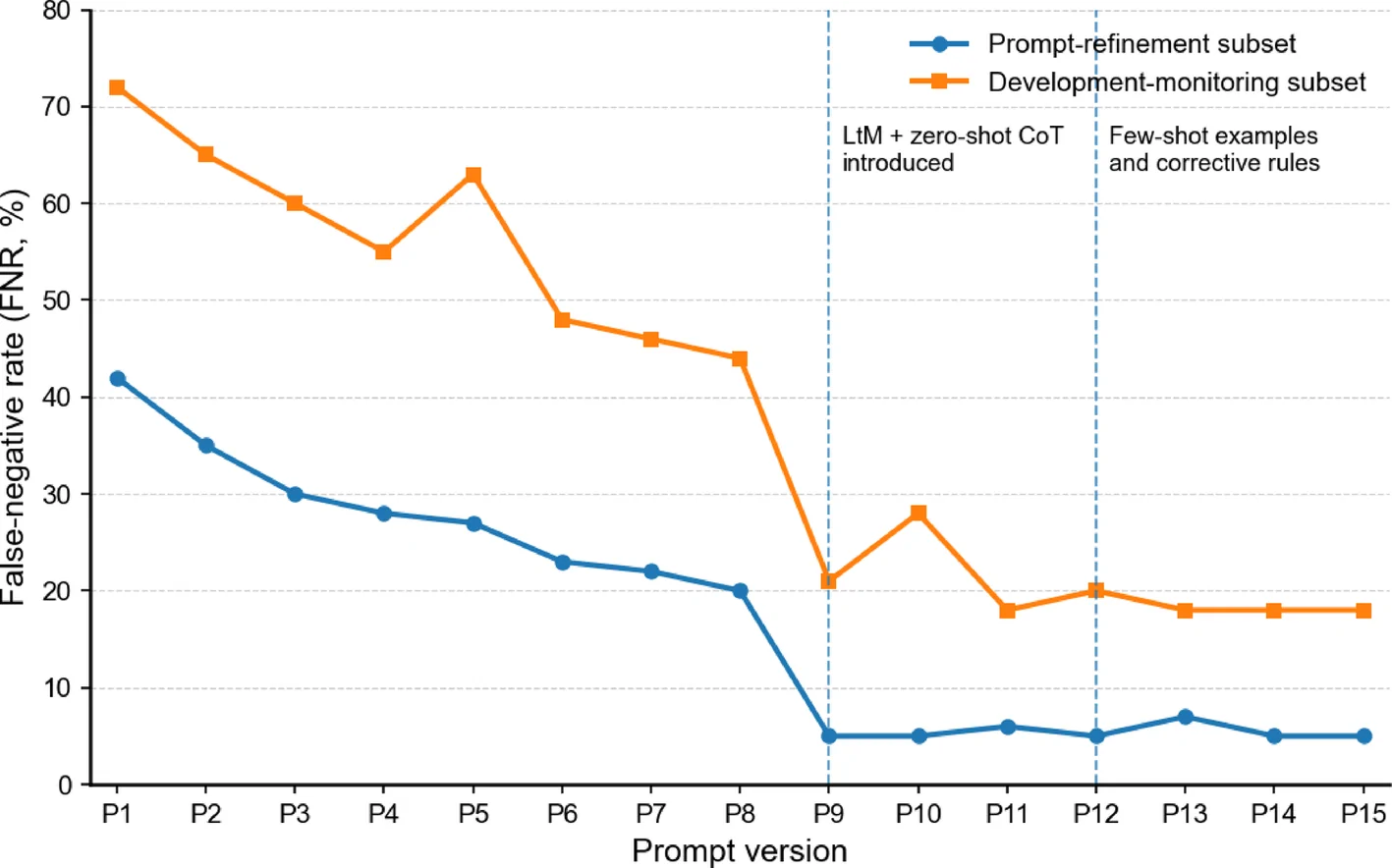
Development-stage FNR across 15 rounds of prompt refinement on PatSAR-Dev-50. The curves show FNR changes across 15 prompt versions in two development-stage subsets within PatSAR-Dev-50. Vertical dashed lines indicate the introduction of major prompt-engineering elements, including least-to-most prompting with zero-shot chain-of-thought instructions at P9 and few-shot examples with corrective rules at P12. FNR was used during prompt development to monitor missed SAR entities rather than to represent parameter-level model training loss

Across 15 rounds of prompt evaluation and error-driven revision on PatSAR-Dev-50, the false-negative rate (FNR) showed an overall downward trend, indicating progressive improvement in extraction completeness during prompt development (Fig. 2). Early versions, P1–P8, which were mainly adapted from prompts for general text analysis, showed relatively high FNR values and recurrent omissions of SAR entities, suggesting limited alignment with the patent-specific extraction task. A marked reduction in FNR was observed after P9, when least-to-most prompting and zero-shot chain-of-thought instructions were introduced, indicating that decomposing SAR extraction into sequential subtasks improved the model’s ability to recover relevant compound–activity information. However, P10 showed increased output variability in repeated analyses of the same task, suggesting that some key terms, extraction boundaries, and expected response formats remained insufficiently specified. After few-shot chain-of-thought examples and more explicit extraction rules were incorporated in P12, the FNR remained within a lower range in subsequent prompt versions. These results suggest that the main improvement occurred after the transition from general extraction prompts to task-specific, constraint-guided prompts, whereas later iterations mainly refined wording and output-format requirements.

Among the later prompt versions, P13 was selected as the finalized prompt. Although P14 and P15 introduced additional detailed constraints, their improvement over P13 was limited, and the added instructions became more closely tied to specific error patterns observed in PatSAR-Dev-50. To avoid excessive tailoring of the prompt to development-stage cases, P13 was fixed as the final version for subsequent evaluation. Thus, the final prompt represented a balance between task-specific guidance and general applicability across patent documents.

To examine the output stability of P13, the finalized prompt was applied in five independent sessions to the same subset of PatSAR-Dev-50. Each trial used the same patent PDFs, finalized prompt, model version, and reference annotations. Across these repeated runs, the coefficient of variation for true-positive counts was 8.69%, whereas false-negative counts showed greater relative variability (Table 4). These results provided descriptive evidence that P13 reduced major recurrent extraction errors and produced relatively stable correctly extracted SAR entities, although variation in missed entities remained.

**Table 4:** Output stability of the finalized prompt in five independent runs

Test round	Total entities	FP	FN	TP
1	1392	6	138	1254
2	1392	0	376	1016
3	1392	5	303	1089
4	1392	0	162	1230
5	1392	0	271	1121
Average			250.0	1142.0
Standard deviation			99.26	99.26
Coefficient of variation			0.3971	0.0869

The finalized prompt P13 was applied five times in independent sessions to the same PatSAR-Dev-50 subset under identical input conditions. TP, FP, and FN denote correctly extracted SAR entities, incorrectly extracted or unsupported SAR entries, and missed reference SAR entities, respectively. The coefficient of variation summarizes variability across repeated runs and was used as a descriptive measure of prompt-output stability

### Core extraction performance in patent SAR analysis

The finalized prompt was evaluated on the core test set of 214 PARP-related patent documents. Across these patents, the final reference annotations contained 8,226 SAR entities, defined as extractable elements contributing to compound-activity relationships, including compound identifiers, structural information, and activity measurements with their corresponding types, values, and units. At the entity level, PatSAR correctly extracted 6,503 entities and missed 1723 entities, corresponding to a recall of 79.05% and an FNR of 20.95%. The model generated 23 false-positive entries, yielding a precision of 99.65% and an F1 score of 88.16% (Table 5). True positives were defined as correctly extracted SAR entities, false negatives as reference SAR entities missed by the model, and false positives as incorrectly extracted non-SAR entries, mismatched SAR relationships, or unsupported structural completions, including IUPAC or structural names not supported by the source patent text.

**Table 5:** Extraction performance on the core patent test set

Target	Entities	TP	FP	FN	Error (%)	Accuracy (%)	Recall (%)	Precision (%)	FNR (%)	F1 score (%)
PARP	8226	6503	23	1723	0.28	99.72	79.05	99.65	20.95	88.16

The finalized prompt was evaluated on PatSAR-Test-214, which contained 214 PARP-related patent PDFs and 8226 expert-annotated SAR entities. TP denotes SAR entities correctly extracted by PatSAR, FP denotes incorrectly extracted non-SAR entries, mismatched compound–activity relationships, or unsupported structural/activity completions, and FN denotes reference SAR entities missed by the model. Recall, precision, FNR, and F1 score were calculated at the SAR-entity level. Error was calculated as FP/(TP + FP + FN), whereas the false-positive rate reported in the text was calculated as FP/(TP + FP), corresponding to 1—precision. Because the open-ended SAR extraction task did not involve a predefined exhaustive set of negative candidate entities, true negatives were not operationally meaningful; therefore, accuracy was reported as 1—error rather than conventional classification accuracy

The performance profile showed a clear imbalance between precision and recall. The high precision indicates that most extracted entries were consistent with the expert reference annotations, suggesting that the finalized prompt constrained the model from introducing many irrelevant compounds or unsupported SAR relationships. In contrast, the lower recall indicates that incomplete extraction remained the main limitation of the workflow. This pattern is consistent with the task design: patent-based SAR extraction requires the model not only to recognize activity values but also to link them correctly with compound identifiers and structural descriptions distributed across different parts of a document.

Most missed entities were associated with document-level extraction challenges rather than incorrect interpretation of explicitly aligned SAR data. Common sources of false negatives included lengthy patent documents, dense multi-page activity tables, activity values embedded outside standard table cells, and incomplete or indirect mappings between compound identifiers and structural descriptions. These cases often required cross-sectional matching between examples, preparation procedures, and activity tables, which remained challenging when explicit textual links were absent or fragmented.

False-positive entries were rare but informative. The 23 false positives mainly involved mismatched activity assignments or unsupported completion of activity fields, including structural or activity information not supported by the source patent text, corresponding to a false-positive rate of 0.35% among model-extracted entities. For example, in patents reporting multiple activity endpoints for the same compound series, the model occasionally transposed values across activity columns or generated an entry for an activity type not supported by the corresponding table cell. These errors show that even when overall precision is high, manual verification remains necessary for complex SAR tables with multiple endpoints or nonstandard layouts.

In addition to standard SAR tables, a subset of extractable SAR entities was located in non-tabular text, such as assay descriptions, examples, preparation notes, or dispersed compound descriptions. In the core test set, 142 confirmed SAR entities were located in such non-tabular contexts. These entries illustrate a practical advantage of the prompt-based workflow: it can search beyond predefined table structures and connect activity information with compound descriptions distributed across narrative sections. This capability is relevant because many conventional automated extraction tools are optimized for structured tables and may not capture SAR information embedded in free text.

### Generalization performance across diverse small-molecule targets

To evaluate whether the finalized prompt could be applied beyond the PARP-focused core test set, we assessed PatSAR on 200 recent patent documents covering ten small-molecule targets: ALK, BTK, CDK, c-MET, EGFR, GLP-1, HER2, IL-17A, KRAS, and PD-L1. The same finalized prompt was used without further target-specific modification.

Across these patents, the reference annotations contained 16,826 SAR entities. PatSAR correctly extracted 14,344 entities and missed 2482 entities, corresponding to an overall recall of 85.25% and an FNR of 14.75%. The model generated 150 false-positive entries, yielding an overall precision of 98.97% and an F1 score of 91.60% (Table 6). This performance was comparable to that observed in the PARP-focused core test set, indicating that the prompt maintained similar extraction behavior across pharmacologically distinct targets.

**Table 6:** Extraction performance on generalization test set

Target	Entities	TP	FP	FN	Error (%)	Accuracy (%)	Recall (%)	Precision (%)	FNR (%)	F1 score (%)
ALK	1425	1248	31	177	2.13	97.87	87.58	97.58	12.42	92.31
BTK	1870	1574	12	296	0.64	99.36	84.17	99.24	15.83	91.09
CDK	1936	1794	8	142	0.41	99.59	92.67	99.56	7.33	95.99
c-MET	1436	1134	11	302	0.76	99.24	78.97	99.04	21.03	87.87
EGFR	1987	1711	18	276	0.90	99.10	86.11	98.96	13.89	92.09
GLP-1	1206	1118	24	88	1.95	98.05	92.70	97.90	7.30	95.23
HER2	2020	1671	8	349	0.39	99.61	82.72	99.52	17.28	90.35
IL-17A	1719	1454	17	265	0.98	99.02	84.58	98.84	15.42	91.16
KRAS	1793	1516	15	277	0.83	99.17	84.55	99.02	15.45	91.22
PD-L1	1434	1124	6	310	0.42	99.58	78.38	99.47	21.62	87.68
Total	16,826	14,344	150	2482	0.88	99.12	85.25	98.97	14.75	91.60

The finalized prompt was applied without target-specific modification to PatSAR-GenTest-200, comprising 200 recent SAR-containing patent PDFs across ten non-PARP small-molecule targets. Entities indicate expert-annotated SAR entities. TP, FP, and FN denote correctly extracted, incorrectly extracted or unsupported, and missed SAR entities, respectively. Performance metrics were calculated at the SAR-entity level for each target and for the overall dataset

**Fig. 3:**
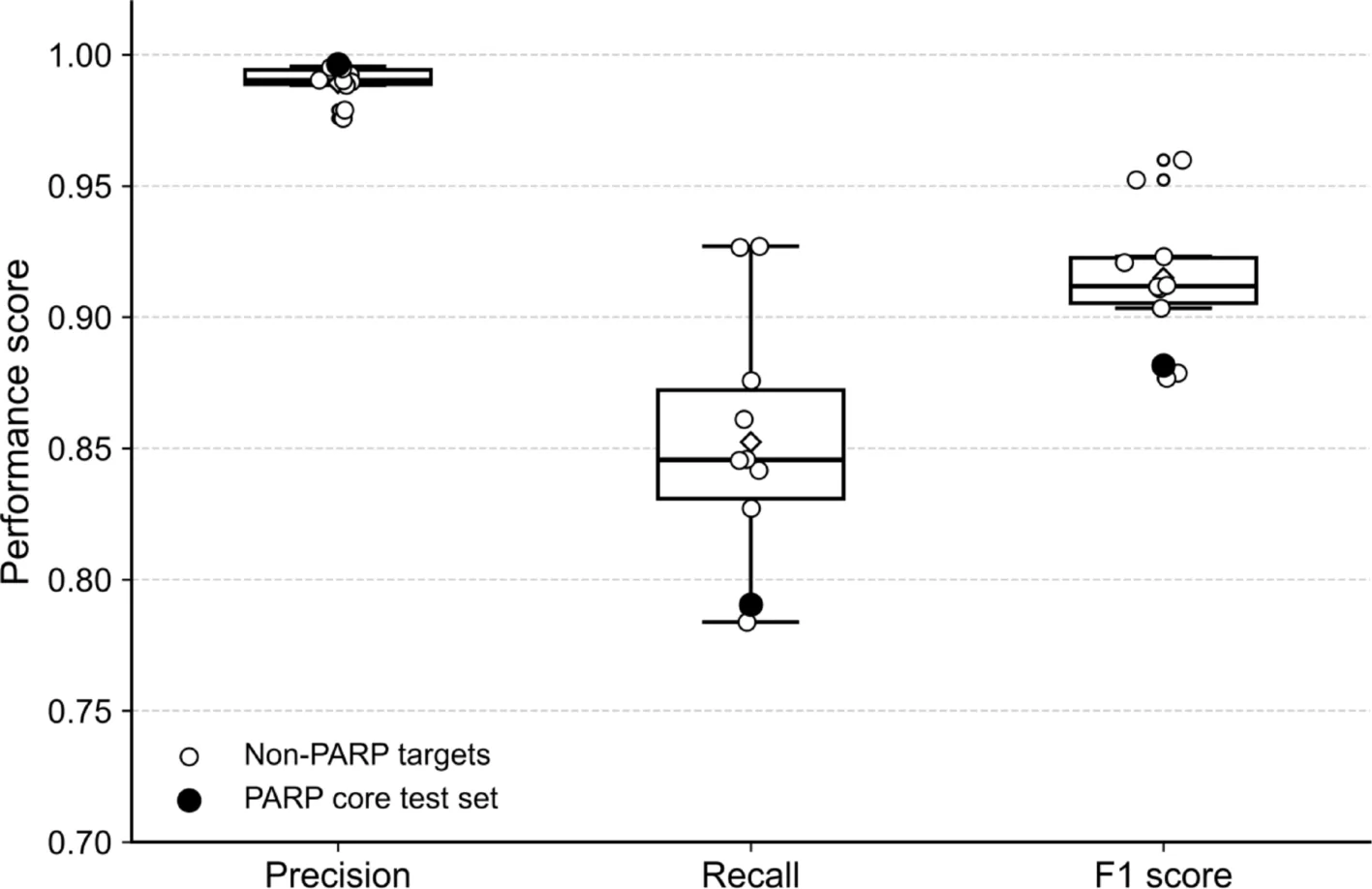
Distribution of target-level extraction performance in PatSAR-GenTest-200 with PARP core-test performance shown for comparison. Boxplots summarize precision, recall, and F1 score across ten non-PARP small-molecule target subsets in PatSAR-GenTest-200. Open circles represent the performance values for individual non-PARP targets. Filled black circles indicate the corresponding PARP performance from PatSAR-Test-214 and are shown as reference points only; they were not included in the boxplot calculations. The center line indicates the median, boxes indicate the interquartile range, whiskers indicate 1.5 × the interquartile range, and diamonds indicate the mean. Scores range from 0 to 1

Performance varied across targets, mainly because of differences in recall rather than precision. Precision remained consistently high across the ten targets, ranging from 97.58% for ALK to 99.56% for CDK. In contrast, recall showed greater target-level variation, ranging from 78.38% for PD-L1 to 92.70% for GLP-1. As a result, target-level F1 scores ranged from 87.68% for PD-L1 to 95.99% for CDK. The distribution of performance metrics further illustrates this pattern: precision was tightly distributed across targets, whereas recall showed wider variability, leading to a broader but still relatively high distribution of F1 scores (Fig. 3).

These results suggest that the finalized prompt was not limited to the PARP patent corpus and could be applied to patents involving different target classes and recent patent-writing styles. However, the variability in recall indicates that extraction completeness remained sensitive to target-specific reporting patterns, document organization, and the way compound–activity relationships were presented. Therefore, the generalization results support cross-target applicability of the workflow while also showing that missed entities remain the principal source of performance variation.

### External comparison

To provide an external quantitative comparison, PatSAR was evaluated on DocSAR-Patent-96, the patent-only subset extracted from the DocSAR-200 benchmark used in the Doc2SAR study. This comparison was conducted using the same Table Recall criterion reported in the original Doc2SAR work. On DocSAR-Patent-96, PatSAR achieved a Table Recall of 81.86%, compared with the reported Doc2SAR result of 74.09% on the patent subset. This corresponded to an absolute improvement of 7.77 percentage points. Because Table Recall requires the molecule and all associated activity values in a SAR table row to be correctly extracted and linked, this improvement indicates that PatSAR recovered a larger proportion of complete compound–activity rows from patent PDFs under the aligned evaluation criterion. This result provides external evidence that the prompt-based PatSAR workflow achieved improved row-level SAR recovery relative to the reported Doc2SAR patent-subset result under the aligned Table Recall criterion.

## Discussion

This study developed and evaluated PatSAR, a prompt-based large language model workflow for extracting structure–activity relationship (SAR) information from patent PDF documents. The central aim was not to train a new model or to build a standalone patent-mining platform, but to examine whether a general-purpose LLM could be guided by task-specific prompt engineering to perform a specialized scientific information extraction task. Across the PARP-focused core test set, the multi-target generalization test set, and the external DocSAR patent subset, PatSAR showed a consistent performance profile characterized by high precision and variable but generally moderate-to-high recall. This indicates that the workflow was effective at limiting unsupported or irrelevant SAR outputs, while incomplete extraction remained the main source of error in complex patent documents.

These results also clarify the intended role of PatSAR. It should be understood as a human–AI curation workflow for converting dispersed patent information into structured SAR tables, rather than as an autonomous replacement for expert review. This distinction is important because patent-derived SAR information often requires interpretation across compound identifiers, structural descriptions, examples, assay tables, and narrative sections. The value of PatSAR therefore lies in assisting expert curation by organizing candidate compound–activity relationships in a structured and auditable format.

Prompt engineering was central to PatSAR because patent SAR extraction requires more than a general instruction to “extract data.” The task requires explicit decisions about what should be considered an SAR entity, how compound identity should be linked to activity endpoints, which chemical entities should be excluded, when unsupported or unavailable information should be marked as “N/A,” and how the extracted information should be formatted for downstream review. Without these task-specific instructions, the model may produce variable table structures, include non-SAR compounds, omit relevant activity values, or complete missing fields without sufficient support from the source patent text [32–34].

Although current LLM systems often include system-level instructions, interface-level optimizations, and built-in capabilities for document processing [24, 25], these general configurations cannot fully specify the requirements of specialized scientific extraction tasks. Built-in prompting can improve general model usability, but it does not define domain-specific entity boundaries, evidence requirements, exclusion rules, or output schemas. User-designed prompt engineering therefore remains important when a task requires specialized definitions and constrained output behavior. In PatSAR, prompt engineering served as a task-specification interface that translated medicinal chemistry requirements into explicit model-executable instructions.

This perspective is important for interpreting the role of prompt engineering in the present study. The prompt did not simply improve the surface format of the model response; it defined the operational rules of the extraction task. The finalized prompt specified SAR entity definitions, compound–activity linkage requirements, exclusion criteria for non-SAR chemical entities, conservative handling of unsupported or unavailable information, and standardized SAR table output. These elements converted patent SAR extraction from an open-ended summarization task into a constrained information extraction workflow.

**Fig. 4:**
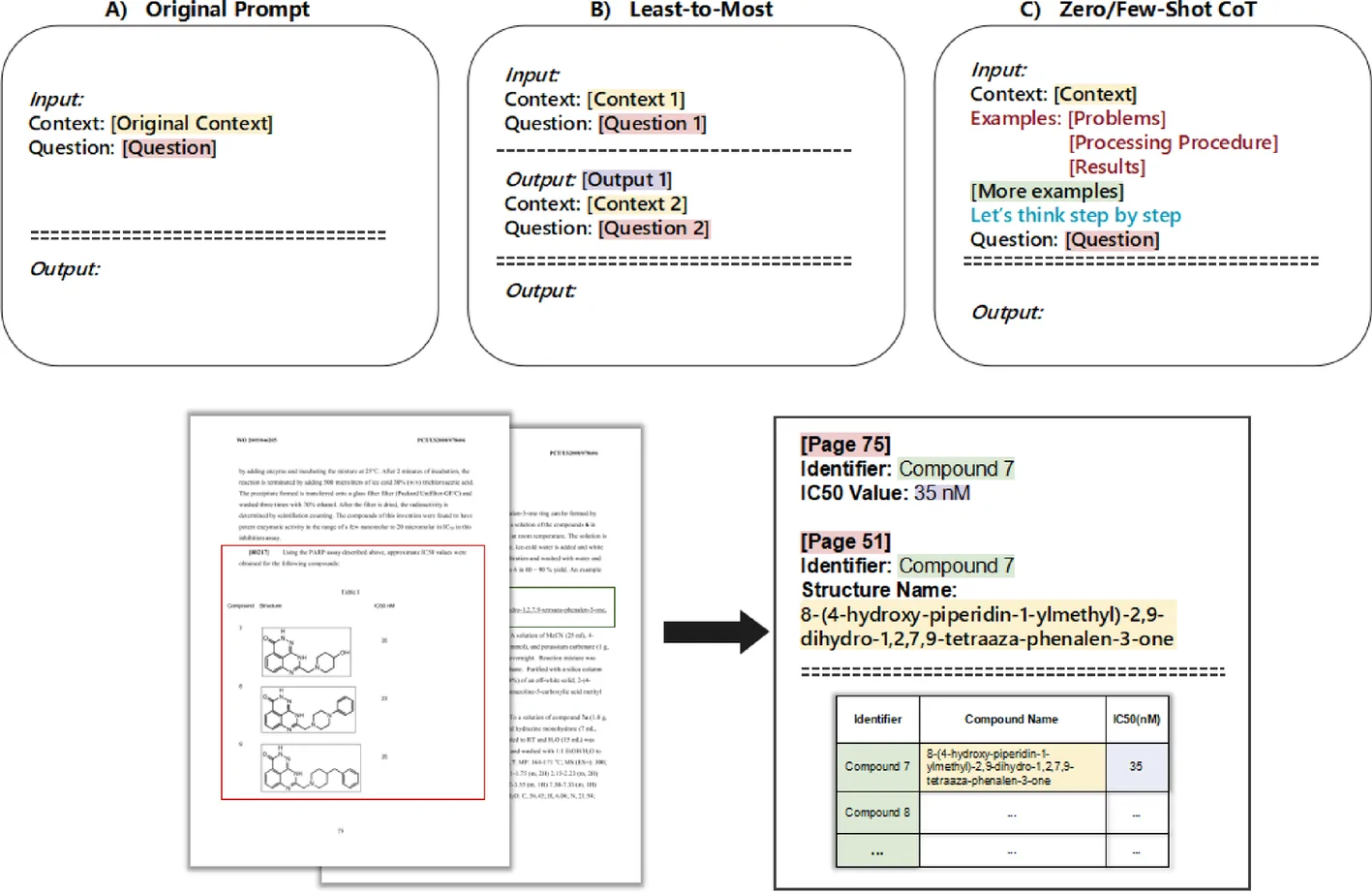
Interaction with the model under different prompt strategies. The upper panels illustrate the transition from a direct original prompt to least-to-most prompting and zero-/few-shot chain-of-thought prompting. The lower panel shows how the refined prompt guided the model to link dispersed activity and structural information from patent PDFs and generate a structured SAR table

The prompt-engineering strategies used in PatSAR are summarized schematically in Fig. 4. The initial direct prompt provided only a broad extraction request, whereas later prompt versions decomposed the task into sequential subtasks and incorporated stepwise checking, few-shot examples, exclusion rules, and structured output constraints. These strategies addressed different failure modes observed during prompt development.

Different prompt strategies addressed different failure modes observed during prompt development. The two-stage design, consistent with least-to-most task decomposition [27], reflected the sequential logic of SAR extraction. During prompt iteration, task decomposition was incorporated according to least-to-most prompting in LLM reasoning, in which a complex task is decomposed into simpler subproblems and solved sequentially. This strategy adapts the broader least-to-most prompting concept originally used in educational and behavioral prompting procedures [28]. In the first stage, the model was instructed to identify compounds associated with reported activity data. In the second stage, it revisited unresolved structural entries by examining examples, preparation procedures, and compound description sections. This ordering was important because activity evidence served as the gatekeeper for inclusion. If the model was first asked to extract all chemical entities, it could include starting materials, intermediates, reagents, solvents, or compounds without reported biological activity. By beginning with compound–activity associations, the workflow reduced the likelihood that non-SAR chemical entities would enter the final table.

Stepwise instructions addressed the cross-sectional nature of patent analysis. In many patent documents, compound identifiers, IUPAC names or structural descriptions, assay endpoints, and activity values are not presented in a single location. A compound may appear in an activity table, its structure may be described in an example, and the assay context may be introduced elsewhere in the document. The model therefore needs to check evidence across tables, examples, preparation procedures, and descriptive sections. In PatSAR, stepwise instructions were not used to claim human-like reasoning, but to encourage sequential verification of compound identity, activity endpoint, activity value, unit, and source support [26, 29]. This helped the model organize dispersed patent content into linked compound–activity records.

Few-shot examples and corrective rules addressed ambiguity in extraction boundaries. During prompt development, some prompt versions produced unstable outputs because the model lacked clear examples of what should be included or excluded. Few-shot examples functioned as boundary-setting demonstrations rather than model-training samples [30]. They clarified when a compound–endpoint association should be counted as an SAR entity, when a non-SAR chemical should be excluded, and when missing structural information should remain “N/A.” Corrective rules also helped reduce unsupported completion by requiring the model to rely on information supported by the patent text rather than generate or infer unsupported IUPAC names, structural descriptions, or activity fields.

Role assignment and explicit constraints provided an additional layer of task framing. The role instruction established a domain-specific context for the task, but the main contribution came from the explicit extraction constraints [39]. These included predefined table columns, preservation of assay labels and units, separation of different activity endpoints, exclusion of non-SAR entities, and conservative handling of unsupported or unavailable information. Together, these constraints shifted the model output away from general patent summarization and toward structured SAR information extraction.

**Fig. 5:**
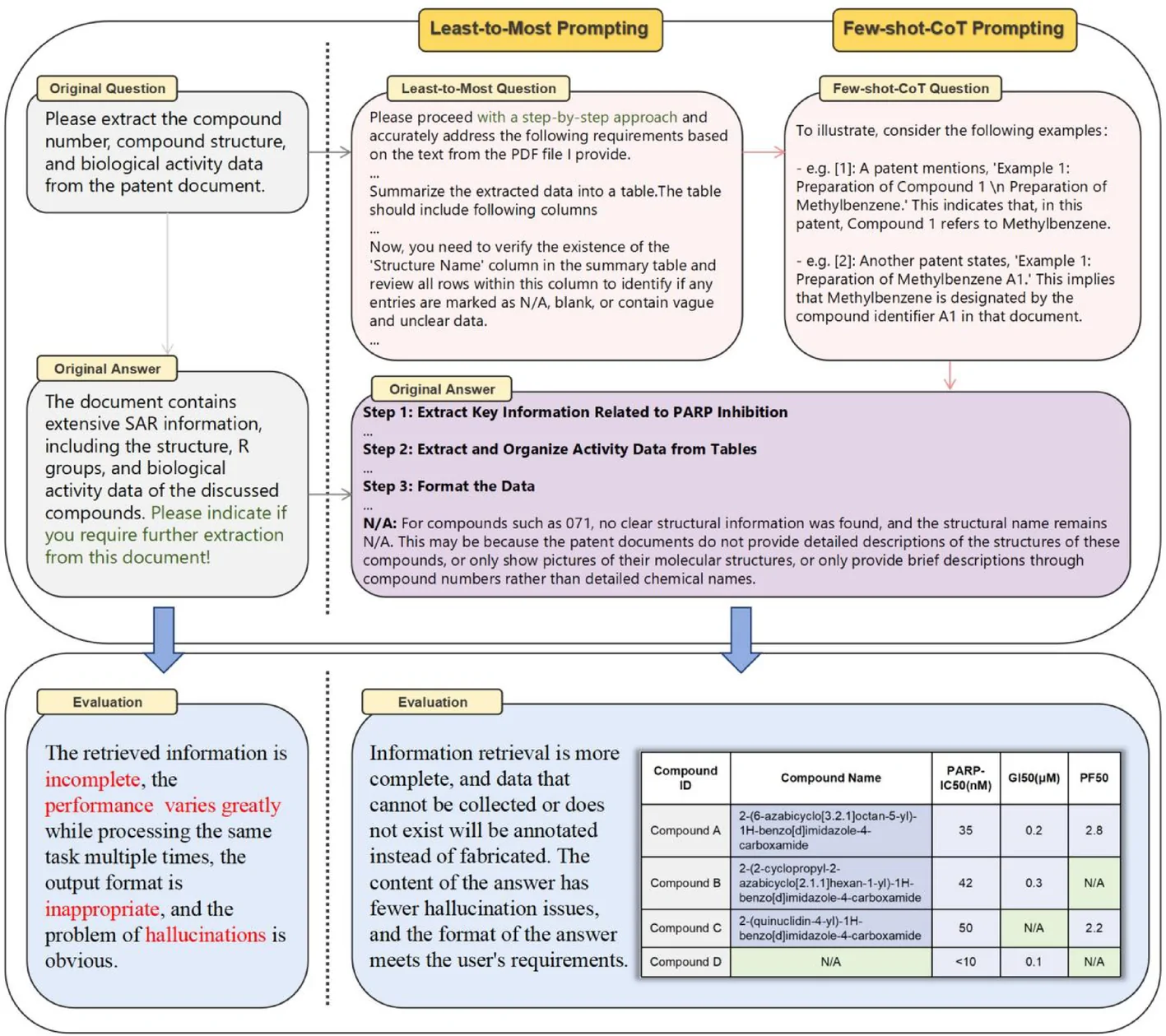
Representative example of model output before and after prompt refinement. The figure illustrates how the initial direct prompt and refined prompt strategies differed in guiding SAR extraction from patent documents. The refined prompt incorporated least-to-most prompting and few-shot chain-of-thought examples, leading to a more structured response with stepwise extraction, conservative “N/A” handling, and standardized SAR table output. This figure is intended as a qualitative illustration of prompt effects rather than a quantitative performance comparison

Representative examples in Fig. 5 illustrate how prompt specification influenced model outputs. With the initial direct prompt, the model was asked to extract SAR-related information but was not given an operational definition of SAR entities, an output schema, inclusion or exclusion criteria, or explicit rules for unavailable information. Therefore, the resulting outputs showed variable table structures, incomplete extraction of activity information, and occasional entries that were not supported by the source text. This observation does not indicate that the model failed under a fully specified instruction; rather, it shows that a broad extraction request is insufficient for a complex patent SAR extraction task.

After prompt refinement, the model output became more consistent because the task was constrained by a predefined SAR table structure, explicit exclusion of non-SAR chemical entities, preservation of activity types and units, and conservative use of “N/A” for unsupported information. The comparison supports the central role of prompt engineering in PatSAR: the prompt did not merely ask the model to “extract data,” but translated the domain task into a sequence of constrained operations that could be applied reproducibly across patent documents.

The Memory function in the ChatGPT environment was used as a persistent layer of task-level instructions. Once configured, these instructions remained available when new patent analyses were initiated, reducing the need to restate the same general task requirements in every chat. These requirements included completeness-oriented extraction, stepwise checking of source evidence, conservative handling of unsupported information, and structured academic-style output. Memory should not be interpreted as model fine-tuning, parameter updating, or an independent extraction algorithm. Rather, it provided persistent contextual guidance for how the task should be performed, while the task-specific extraction behavior was primarily controlled by the finalized prompt. The detailed Memory configuration is provided in the Supplementary Materials.

PatSAR should be understood as a prompt-based workflow implemented through the ChatGPT web interface. Users with access to the selected LLM interface can apply the workflow by using the finalized prompt and uploading patent PDFs for document-by-document analysis. This implementation does not require local model deployment, but access remains dependent on login requirements, model availability, file-upload constraints, and platform-specific usage limits.

In practical use, PatSAR can serve as an upstream curation-assistance tool for transforming patent PDFs into preliminary SAR tables. Such tables can support patent landscape review, early-stage SAR curation, scaffold comparison, compound–activity data organization, and preparation of structured datasets for downstream medicinal chemistry analysis. The workflow is particularly suitable for reducing the manual burden of locating and organizing compound–activity records before expert verification, rather than for making final SAR judgments independently.

Several limitations should be considered when interpreting this study. First, PatSAR depends on proprietary LLM interfaces. Model availability, context-window size, file-processing behavior, memory functions, and output style may change over time as providers update or retire deployed models. In this study, all main performance metrics were computed using ChatGPT-5 to ensure consistency across the core test set, generalization test set, and external comparison set. Nevertheless, the same prompt may not perform identically across future model versions, other proprietary LLMs, or open-source models. Therefore, future applications should document the model version, interface configuration, and file-processing conditions, and should revalidate the workflow when the underlying model or platform changes.

Second, the current study did not perform a full head-to-head comparison across candidate LLMs such as Claude, Gemini, Kimi, and open-source models. External document-analysis rankings were used only as a screening reference for selecting an implementation platform, not as a task-specific benchmark for patent SAR extraction. Future work should evaluate the same prompt framework across multiple models under identical patent-level evaluation conditions.

Third, the evaluation datasets focused on patents containing explicit SAR-relevant information. Patents or documents without actionable SAR data were excluded during dataset construction, and therefore the present study did not estimate the false-positive rate on irrelevant or no-SAR documents. Future work should include negative-control patents lacking SAR information to quantify spurious SAR extraction and determine whether an upstream document-relevance screening step is needed.

Fourth, PatSAR was evaluated as a document-by-document workflow. Simultaneous multi-document or batch processing was not assessed. The current implementation also has scalability limitations because users must identify, obtain, and submit relevant patent PDFs before extraction. In its present form, PatSAR is most suitable for medicinal chemists, patent analysts, cheminformatics researchers, or domain experts who already have candidate patent documents and need to convert dispersed SAR information into structured tables for review. Although batch analysis may be technically possible depending on interface limits and context-window capacity, combining multiple patents in a single model context could introduce cross-document interference or reduce extraction completeness. Future systems may integrate the prompt-based extraction workflow with patent databases, automated retrieval tools, document-relevance screening, and batch-processing pipelines to support larger-scale patent landscape analysis.

Finally, the current workflow remains sensitive to document quality and organization. Missed entities were more likely when patents were lengthy or contained dense multi-page tables, activity values outside standard table cells, incomplete compound–activity mappings, fragmented structural descriptions, or low-quality PDF content. Future systems may benefit from integrating LLM prompting with specialized table parsers, chemical structure recognition tools, document preprocessing and OCR pipelines, and human-in-the-loop review interfaces. Expanding evaluation to additional targets, jurisdictions, languages, patent statuses, and no-SAR controls will further clarify the generalizability and practical limits of prompt-based SAR extraction from patents.

## Supplementary Information

Below is the link to the electronic supplementary material.

Supplementary Material 1

## Data Availability

The patent documents analyzed in this study are publicly available from the European Patent Office, Google Patents, and the original sources associated with the DocSAR-200 benchmark where applicable. The internally constructed PatSAR datasets generated in this study, including PatSAR-Dev-50, PatSAR-Test-214, PatSAR-GenTest-200, and the model-consistency subset, are deposited in Zenodo under the accession code https://doi.org/10.5281/zenodo.20482853. The persistent URL is: https://zenodo.org/records/20482853. These files include the patent documents and dataset metadata used for prompt development, core evaluation, generalization testing, and model consistency analysis. The code and prompt materials associated with this study are available in the GitHub repository: https://github.com/Clausius2000/PatSAR-Extracting-Structure-Activity-Relationships-from-Patents-with-Large-Language-Models. The repository includes the random-selection script used for constructing PatSAR-GenTest-200, the complete prompt-development record from P1 to P15, the finalized PatSAR prompt, and related supplementary materials needed to inspect and reproduce the prompt-based workflow.
