# Supplementary material for: Extracting structure–activity relationships from patents with large language models
Source: BMC Bioinformatics. 2026 Aug 3;27:251. doi: 10.1186/s12859-026-06587-w (PMC13721409; doi:10.1186/s12859-026-06587-w)
Supplement: Supplementary file 1 [file 12859_2026_6587_MOESM1_ESM.docx]

**Supplementary Materials**

**Table S1.** Jurisdiction distribution of datasets.

| **Datasets** | **Jurisdiction** | **n** | **Percentage (%)** |
| --- | --- | --- | --- |
| PatSAR-Dev-50 | WO | 29 | 58.00 |
|  | CN | 20 | 40.00 |
|  | US | 1 | 2.00 |
| PatSAR-Test-214 | WO | 182 | 85.05 |
|  | US | 8 | 3.74 |
|  | JP | 6 | 2.80 |
|  | CN | 5 | 2.34 |
|  | EP | 5 | 2.34 |
|  | CA | 3 | 1.40 |
|  | AR | 2 | 0.93 |
|  | TW | 2 | 0.93 |
|  | KR | 1 | 0.47 |
| PatSAR-GenTest-200 | CN | 72 | 36.00 |
|  | WO | 55 | 27.50 |
|  | US | 20 | 10.00 |
|  | TW | 12 | 6.00 |
|  | EP | 10 | 5.00 |
|  | CA | 9 | 4.50 |
|  | JP | 7 | 3.50 |
|  | AU | 6 | 3.00 |
|  | KR | 6 | 3.00 |
|  | ES | 2 | 1.00 |
|  | RU | 1 | 0.50 |

**Table S2.** Publication-status distribution of datasets.

| **Datasets** | **Document type inferred** | **n** | **Percentage (%)** |
| --- | --- | --- | --- |
| PatSAR-Dev-50 | PCT application publication | 29 | 58.00 |
|  | Application publication | 21 | 42.00 |
| PatSAR-Test-214 | PCT application publication | 182 | 85.05 |
|  | Application publication | 15 | 7.01 |
|  | Granted patent publication | 17 | 7.94 |
| PatSAR-GenTest-200 | PCT application publication | 55 | 27.50 |
|  | Patent application publication | 106 | 53.00 |
|  | Granted patent publication | 39 | 19.50 |

**Table S3.** IPC and CPC subclasses represented in datasets.

| **Datasets** | **Classification system** | **Represented subclasses** |
| --- | --- | --- |
| PatSAR-Dev-50 | IPC | A61K, A61P, C07D, C07F, C07K, G01N |
|  | CPC | A61P, C07D, A61K, C07B, C07K, C07F, G01N |
| PatSAR-Test-214 | IPC | C07D, A61K, A61P, C07C, G01N |
|  | CPC | A61P, C07D, A61K, C07C, C07B, G01N |
| PatSAR-GenTest-200 | IPC | A61P, A61K, C07D, C07K, C12N, C07F, C12Q, G01N |
|  | CPC | A61P, A61K, C07D, C07K, C12N, C07F, C07B, G01N |

*Only major drug-, chemistry-, and bioactivity-relevant subclasses are shown.

**Table S4.** Major assignees or applicants represented in PatSAR datasets.

| **Datasets** | **Harmonized assignee/applicant** | **n** |
| --- | --- | --- |
| PatSAR-Dev-50 | GENFLEET THERAPEUTICS SHANGHAI INC | 5 |
|  | INVENTISBIO CO LTD | 3 |
|  | JIANGSU HENGRUI MEDICINE CO | 3 |
|  | MEDSHINE DISCOVERY INC | 3 |
|  | ABBISKO THERAPEUTICS CO LTD | 2 |
|  | BEIGENE LTD | 2 |
|  | PFIZER | 2 |
| PatSAR-Test-214 | JANSSEN PHARMACEUTICA NV | 10 |
|  | ANGELETTI P IST RICHERCHE BIO | 10 |
|  | JONES PHILIP | 10 |
|  | ABBOTT LAB | 9 |
|  | PENNING THOMAS D | 9 |
|  | KUDOS PHARM LTD | 8 |
|  | BASF AG | 7 |
| PatSAR-GenTest-200 | BEIGENE LTD | 4 |
|  | DAIICHI SANKYO CO LTD | 4 |
|  | ASTRAZENECA UK LTD | 4 |
|  | MINDRANK AI LTD | 3 |
|  | RELAY THERAPEUTICS INC | 3 |
|  | SHANGHAI HANSOH BIOMEDICAL CO LTD | 3 |
|  | LEI BAILIN | 3 |
|  | INCYTE CORP | 3 |
